# Determination of Inherent Dissolution Performance of Drug Substances

**DOI:** 10.3390/pharmaceutics13020146

**Published:** 2021-01-22

**Authors:** Dominik Sleziona, Amelie Mattusch, Gerhard Schaldach, David R. Ely, Gabriele Sadowski, Markus Thommes

**Affiliations:** 1 Laboratory of Solids Process Engineering, Faculty of Biochemical and Chemical Engineering, TU Dortmund, Emil-Figge-Str. 68, 44227 Dortmund, Germany; dominik.sleziona@tu-dortmund.de (D.S.); amelie.mattusch@tu-dortmund.de (A.M.); gerhard.schaldach@tu-dortmund.de (G.S.); 2 School of Advanced Manufacturing, Engineering and Applied Science, Ivy Tech Community College Lafayette, 3101 S Creasy Ln, Lafayette, IN 47905, USA; dely7@ivytech.edu; 3 Laboratory of Thermodynamics, Faculty of Biochemical and Chemical Engineering, Technical University Dortmund, Emil-Figge-Str. 70, 44227 Dortmund, Germany; gabriele.sadowski@tu-dortmund.de

**Keywords:** drug release, flow channel, intrinsic dissolution, surface reaction, two-film theory

## Abstract

The dissolution behavior of novel active pharmaceutical ingredients (API) is a crucial parameter in drug formulation since it frequently affects the drug release. Generally, a distinction is made between surface-reaction- and diffusion-controlled drug release. Therefore, dissolution studies such as the intrinsic dissolution test defined in the pharmacopeia have been performed for many years. In order to overcome the disadvantages of the common intrinsic dissolution test, a new experimental setup was developed within this study. Specifically, a flow channel was designed and tested for measuring the mass transfer from a flat, solid surface dissolving into a fluid flowing over the surface with well-defined flow conditions. A mathematical model was developed that distinguishes between surface-reaction- and diffusion-limited drug release based on experimental data. Three different drugs—benzocaine, theophylline and griseofulvin—were used to investigate the mass flux during dissolution due to surface reaction, diffusion and convection kinetics. This new technique shows potential to be a valuable tool for the identification of formulation strategies.

## 1. Introduction

The low aqueous solubility of new active pharmaceutical ingredients (API) has been an issue in pharmaceutical development for many years [1]. Therefore, several formulation concepts have been proposed to overcome the low bioavailability of poorly water-soluble drugs in oral administration [2]. In order to identify effective formulation strategies, physical–chemical properties such as solubility and permeability have been considered [3]. The biopharmaceutical classification system has been used in this respect [4]. Presently, particle properties such as size, shape and powder flow are also considered in drug formulation in the manufacturing classification system [5,6].

For many years, drug dissolution has been described by diffusion models, such as Noyes–Whitney [7] and Hixson–Crowell [8]. Later approaches such as those developed by Higuchi [9] and Siepmann [10] as well as Göpferich, Langer [11] and Hasa [12] are based on Fick’s law [13]. More recently, drug–solvent interactions have been shown to affect drug release in addition to diffusion [14,15,16]. This has been adopted from other research fields, where this phenomenon is already known [17,18,19,20,21].

**Figure 1:**
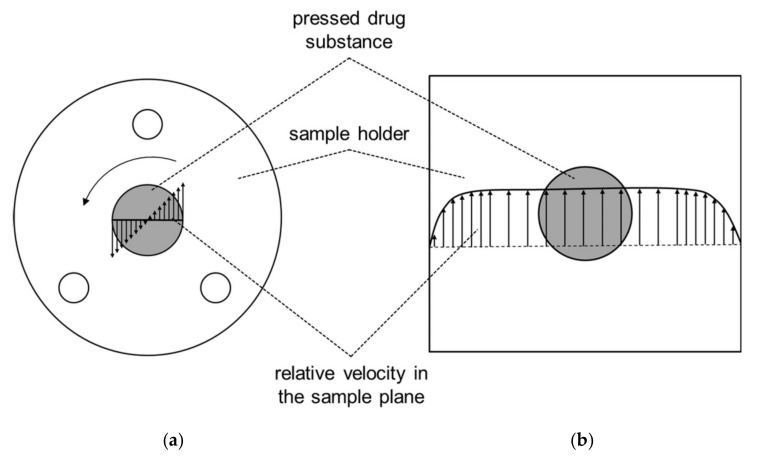
Relative velocity vectors for intrinsic dissolution (PhEur 9.0 chapter 2.9.29) (**a**) and the novel flow channel (**b**).

In order to characterize the dissolution performance of drug substances, the intrinsic dissolution test (PhEur 9.0 method 2.9.29) was considered [22]. In addition to the limitations already known in the literature [23], other issues such as gas bubble formation and inconsistent flow fields limit its usefulness. The gas bubbles have three origins: 1. incorporation during the vertical construction of the rotating disc system, 2. remaining gas leaving the solvent, and 3. gas escaping from the tapped holes of the sample holder due to thermal expansion. These gas bubbles are able to occupy parts of the release area, which leads to variability in the dissolution rate of the drug substance. Additionally, they can disturb the hydrodynamics. Moreover, there is a velocity gradient at the surface of the sample (Figure 1a), caused by the tip speed of the rotating cylinder, which is an inherent limitation of the intrinsic dissolution method.

As a result, the molecules at the surface experience different mechanical stresses depending on their position, radially. This could lead to different release mechanisms at the sample surface, causing variability in the dissolution rate.

The aim of this study was to develop a method in which the flow conditions were well defined and reproducible (Figure 1b). This was realized by suffusing a flat surface of a drug sample in a laminar flow field, which enabled the collection of physically meaningful diffusion data and surface reaction effects during the dissolution of the drug substance.

## 2. Materials and Methods

### 2.1. Materials

The investigated active pharmaceutical ingredients, benzocaine acetate (Caser&Loretz, Hilden, Germany), theophylline monohydrate (BASF, Ludwigshafen, Germany) and griseofulvin (Hawkins, Roseville, MN, USA), are often reported in the literature as model drug substances. Deionized water was used as the dissolution solvent for all experiments.

### 2.2. Design of the Flow Channel

Computational fluid dynamics (CFD) software (Ansys CFX 19.1, Ansys, Inc., Canonsburg, PA, USA) was used to design the flow channel geometry. Different geometries were tested and optimized until the optimal geometry was found as discussed (Section 3.1). Starting with the schematic drawing, the volume of the channel was extracted and discretized with a hexahedral mesh with a maximum side length of 1.0 mm. The computational domain consisted of about 420,000 control volumes. For the numerical experiments, an inlet velocity of the fluid corresponding to a certain Reynolds number was used for the inlet boundary condition. A pressure boundary was set for the outlet. In order to capture turbulences, which occur in the entrance of the flow channel, the “shear-stress transport model” [24,25] was used. Residuals lower than 10^−6^ were established as the primary criterion for convergence. All calculations were performed in parallel mode on a workstation with ten cores. The computational time for each Reynolds number was about 2 hours on a desktop computer.

### 2.3. Dissolution Experiments in the Flow Channel

One gram of powder was compressed in a 13 mm-diameter die using a hydraulic press (Type 3, Paul Weber, Bösingen, Germany) with a compression force of 10 kN for about 30 s. Afterwards, the die was inserted in the flow channel, which was attached to a 700 mL reservoir (25 °C, pH ~ 6.5). During the measurement, the pump speed (BVP-Z 1830, Ismatec, Wertheim, Germany) was adjusted to realize various velocities within the flow channel, resulting in predefined Reynolds numbers. The drug concentration was measured by UV spectroscopy (10 mm cuvette from Lambda 25, PerkinElmer, Inc., Waltham, USA; 50 mm cuvette from Specord 200 Plus, Analytik Jena AG, Jena, Germany). Afterwards, the flux from the sample surface was calculated.

## 3. Results and Discussion

### 3.1. Experimental Setup

A flow channel was designed in order to realize defined flow conditions across the flat sample surface, where ideal wetting conditions were assumed. From the hydrodynamic point of view, a circular flow channel was preferred as demonstrated by Missel et al. [26]. However, it was impractical to prepare a curved sample surface. Therefore, a rectangular flow channel was utilized [21,27,28]. The flow conditions were chosen based on the intrinsic dissolution setup of the pharmacopeia. Typical rotation speeds are between 50 and 150 rpm, with a sample diameter of 8 mm, which corresponds to Reynolds numbers between 83 and 250 at half a radius from the center of the tablet (2 mm). In order to assess the influence of the Reynolds number on the drug release, the flow channel was designed to be able to vary the Reynolds number up to 250. Related to the flow channel geometry, the Reynolds number could be calculated according to

(1) $$Re=\frac{v\cdot D_{hydr}\cdot \rho_{l}}{\eta }=\frac{\frac{\dot{V}}{a\cdot b}\cdot \frac{4\cdot A}{U}\cdot \rho_{l}}{\eta }$$

Here, *Re* is the Reynolds number, *v* is the fluid velocity, *D_hydr_* is the hydraulic diameter, *ρ_l_* is the fluid density, *η* is the dynamic viscosity, $\dot{V}$ is the volume flow rate, *a* is the width, *b* is the height, *A* is the area and *U* is the circumference of the flow channel.

The inlet length of the flow channel, *l_inlet_*, was estimated [29] in order to obtain laminar flow conditions at the sample position (Equation (2)), already proposed by Greco et al. [30] and Boetker et al. [31], as follows:

(2) $$l_{inlet}=0.06\cdot Re\cdot D_{hydr}$$

However, according to the estimation of Ward-Smith [32], the fluid flow profile is mainly influenced by the aspect ratio (width/height) of the flow channel, and five was advised for a laminar flow without a velocity gradient. Such geometries have been used successfully in other studies [33,34]. Therefore, a 50 mm-wide and 10 mm-high channel was used to house the 13 mm sample holder.

In addition to the dimensions of the flow channel in the vicinity of the sample, parameters such as the length of the flow channel as well as the inlet and outlet dimensions had to be defined. These parameters were determined from fluid dynamics simulations (Section 2.2), which were conducted to optimize the flow channel geometry for laminar fluid flow over the sample.

**Figure 2:**
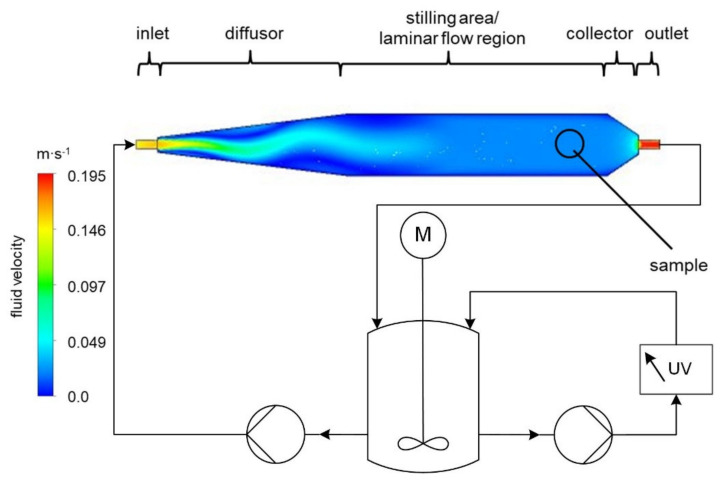
Experimental setup of the flow channel dissolution apparatus for a Reynolds number of 250.

**Figure A1:**
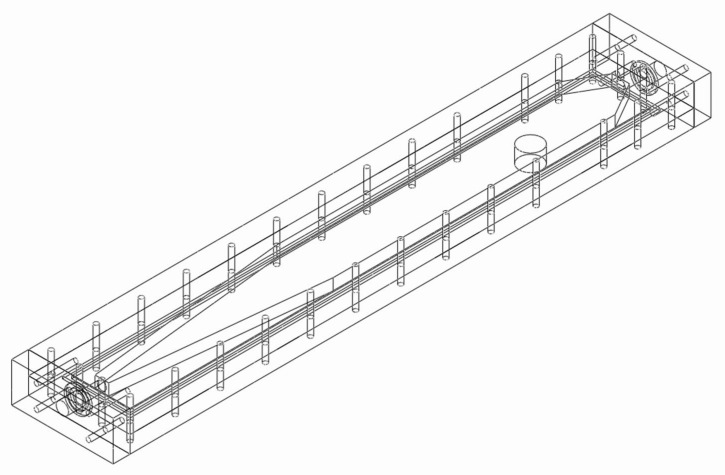
CAD drawing of the used flow channel.

The resulting flow channel consisted of a 12 mm-diameter cylindrical inlet port on a 181 mm-long conical diffusor, followed by a 245 mm-long stilling area/laminar flow region terminated by a 5.8 mm-long collector region with a 12 mm-diameter cylindrical outlet port (Figure 2). An isometric view of the flow channel is provided in Figure A1 in the Appendix A and a construction view in the Supplementary Materials (Figure S1).

**Figure 3:**
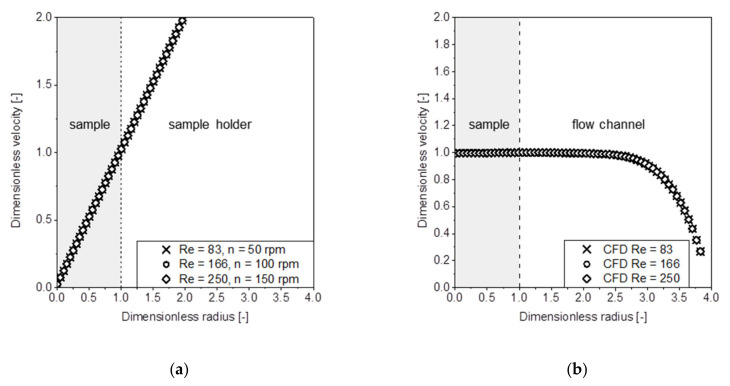
Computational fluid dynamics (CFD) simulation of the dimensionless velocity $\left(\frac{v_{i}}{v_{max}}\right)$as a function of the dimensionless sample radius $\left(\frac{r_{i}}{r_{sample}}\right)$ for the intrinsic dissolution method (**a**) and laminar flow channel (**b**), where *r_i_* = 0 is the sample center.

In addition to the Reynolds number (Equation (1)), the velocity profile at the sample surface was evaluated based on CFD data. Since the velocity varies with the Reynolds number, the velocity profile was normalized to the maximum velocity in the center of the flow channel. The velocity profiles for the intrinsic dissolution as well as the flow channel setup are shown in Figure 3.

In the intrinsic dissolution setup, the relative velocity varies with respect to the radius of the sample. This leads to differences in the dissolution rate with respect to the position on the sample as described previously.

This variation of the dissolution rate across the sample due to the non-constant velocity profile is the main disadvantage of the pharmacopeia method [35]. By contrast, the variation of the velocity profile across the sample surface in the proposed flow channel (Figure 2) is nearly zero. Therefore, each position on the surface is subjected to the same flow conditions and should therefore exhibit the same drug dissolution behavior.

### 3.2. Measuring Protocol

**Figure 4:**
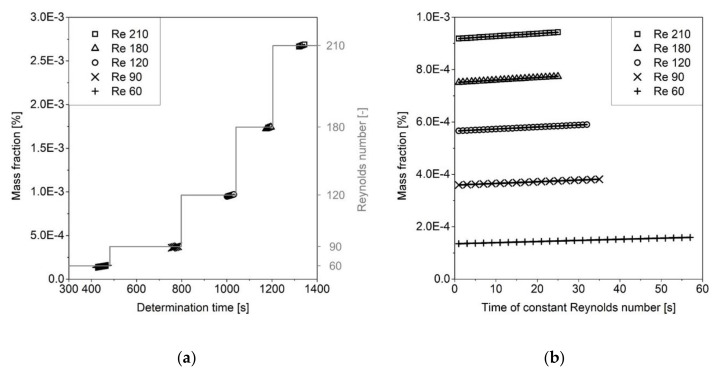
Theophylline dissolution kinetics at different Reynolds numbers (**a**) and regressions of the release rates (**b**).

During a single determination, the pump speed was varied in order to obtain Reynolds numbers from 60 to 210 (Figure 4a). After an equilibration period, the drug release was measured as a function of time by inline UV spectroscopy. Constant release rates were obtained for all Reynolds numbers since sink conditions were applied (Figure 4b). Each determination was performed at least six times for each substance.

The horizontal steps in Figure 4a correspond to the Reynolds numbers shown on the secondary axis for which each set of measurements was made. The primary axis shows the mass fraction dissolved as a function of time over the course of the experiment as the Reynolds number was successively increased. Figure 4b shows the release as a function of time for the individual Reynolds numbers. Sink conditions were maintained for the entire experiment.

### 3.3. Modeling

In order to elaborate the dissolution behavior of a substance further, a model had to be derived that captured the surface reaction kinetics as well as the diffusion kinetics. Therefore, the drug release rate was normalized by the surface area of the sample to calculate the flux from the surface into the liquid. Throughout the study, a mass flux ($J^{w}$) was used in favor of the molar flux and is described below.

**Figure 5:**
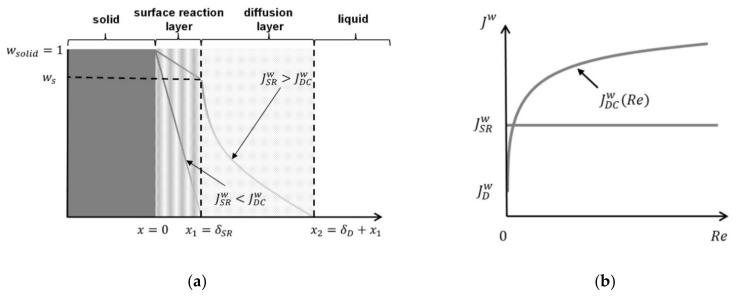
(**a**) Schematic representation of the dissolution process in terms of a diffusion zone and a surface reaction zone. (**b**) Corresponding mass flux $J^{w}$as a function of the Reynolds number.

The model was based on the two-film theory of Whitman [36], which models the surface of the solid as two distinct but adjacent layers (Figure 5a). In the first layer, the molecules are dissolved from the solid surface into the liquid phase, which is called the surface reaction. In the adjacent layer, the dissolved molecules diffuse into the bulk liquid. At steady state, the thicknesses of these layers (*δ_D_* and *δ_SR_*) as well as the total flux are constant. The dissolution rate is limited by either the surface reaction ($J_{SR}^{w}$) or the diffusion ($J_{D}^{w}$), depending on the substance properties and dissolution conditions. An increase in the Reynolds number could decrease the thickness of the diffusion layer (*δ_D_*) and increase the dissolution rate for a diffusion-limited system, which can be considered as convection ($J_{DC}^{w}$, Figure 5b).

The basis of the new model is Fick’s first law (Equation (3)). According to this, the molar flux (J) is a linear function of the gradient of the chemical potential *(∂µ/∂x*).

(3) $$J=-k\cdot {\left(\frac{\partial \mu }{\partial x}\right)}_{p,T}$$

Assuming that the gradient in chemical potentials equals the concentration gradient and making several other assumptions discussed in detail by Atkins et al. [37], the molar flux can be expressed as a linear function of the concentration gradient (*dc*/*dx*), which was the original definition of Adolf Fick [13]. The diffusion coefficient *D* represents the correlation between the concentration gradient and the molar flux as follows:

(4) $$J=-D\cdot \left(\frac{dc_{i}}{dx}\right)$$

The concentration gradient was reformulated in terms of a weight fraction (*w_i_*) gradient, which was more convenient for simplifying the model later on. Herein, the weight fraction in the solid is one, *ρ_total_* is the overall liquid density, and *M_i_* is the molar weight of the diffusing species, so Equation (4) becomes

(5) $$J=-\frac{D\cdot \rho_{total}}{M_{i}}\cdot \left(\frac{dw_{i}}{dx}\right)$$

Multiplying both sides by the molar weight allows Equation (5) to be expressed as a mass flux $J^{w}$[38] (Equation (6)). The flux is defined as the number of molecules leaving the solid surface going into the liquid per unit time. Since the actual experiment measures the number of molecules appearing in the liquid, the negative sign was removed for the purpose of this discussion.

(6) $$J^{w}=D\cdot \rho_{total}\cdot \left(\frac{dw_{i}}{dx}\right)$$

The mass flux into the diffusion layer due to the surface reaction can be expressed in a similar fashion to the diffusion of the solute into the bulk solvent described by Equation (6). In terms of diffusion-controlled dissolution ($J_{SR}^{w}>J_{DC}^{w}$), the mass flux is described by Equation (7). Thus, the solution on the solid side of the diffusion layer is saturated (*w* = *w_s_*), while the solvent on the opposite side of the diffusion layer has a weight fraction of nearly 0 under sink conditions. At steady state, the weight fraction gradient driving diffusion across the diffusion layer is *w_s_/δ_D_*. For drugs with low solubility, the density of the liquid (*ρ_l_*, the density of the saturated solution) is close to the density of the pure solvent, so

(7) $$J_{D}^{w}=\frac{D\cdot \rho_{l}\cdot w_{s}}{\delta_{D}}$$

Therefore, the flux into the solvent for diffusion-controlled drug release under steady-state, sink conditions is constant as well. However, the thickness of the diffusion layer (*δ_D_*) is a function of the relative velocity of the fluid and, therefore, the Reynolds number (*Re*). Convection must therefore be considered. According to Murzin and Salmi [39], convection can be considered as a flux increase relative to diffusion. The magnitude of the convective flux is a function of the Sherwood number (*Sh*), which is a function of the Reynolds number and is defined as the ratio between the convectional and diffusional transport rates. The flux resulting from both diffusion and convection is given by

(8) $$J_{DC}^{w}=Sh\left(Re\right)\cdot J_{D}^{w}+J_{D}^{w}$$

This proposed dissolution model was developed based on the work of Pohlhausen [40], who described the Sherwood correlation for a laminar flow over a flat surface.

(9) $$Sh=0.664\cdot Re^{\frac{1}{2}}\cdot Sc^{\frac{1}{3}}$$

In this empirical equation, the Sherwood number is expressed as a function of the Reynolds as well as the Schmidt number (*Sc*). Substituting Equations (7) and (9) into Equation (8) yields

(10) $$J_{DC}^{w}=0.664\cdot {\left(\frac{\eta }{\rho_{l}\cdot D}\right)}^{\frac{1}{3}}\cdot Re^{\frac{1}{2}}\cdot \frac{D\cdot \rho_{l}\cdot w_{s}}{\delta_{D}}+\frac{D\cdot \rho_{l}\cdot w_{s}}{\delta_{D}}$$

This linear model can be used to describe experimental data from the flow channel, where the Reynolds number is varied, and the total mass flux is measured. Based on the Stokes–Einstein equation [41,42], the diffusion coefficient can be calculated as follows

(11) $$D=\frac{k_{B}\cdot T}{6\cdot \pi \cdot r_{mol}}$$

where *k_B_* is the Boltzmann constant, *T* is the absolute temperature and *r_mol_* is the radius, which can be derived via the molar volume and Avogadro’s constant. This enables Equation (10) to be solved for the diffusion layer thickness (*δ_D_*) at *Re* = 0, which is the only parameter that has to be fitted to experimental data. The model is capable of describing diffusion as well as convection in the newly designed flow channel for substances exhibiting diffusion-controlled drug release as well as for substances exhibiting surface-reaction-limited drug release.

For $J_{SR}^{w}>J_{DC}^{w}$, the system is diffusion limited, because the surface reaction layer is always at the solubility limit. The flux for pure diffusion with no convection is $J_{D}^{w}$ and increases with an increasing Reynolds number due to the reduction of the diffusion layer thickness *δ_D_*. The system becomes surface reaction limited when the mass flux $J_{SR}^{w}$ cannot be increased by increasing *Re* (Figure 5b). Thus, for $J_{SR}^{w}<J_{DC}^{w}$, the system is surface reaction limited, because the diffusion layer is depleted more rapidly than the solid can be dissolved. In other words, when the mass flux in the flow channel is independent of the Reynolds number, the system will be surface reaction limited ($J_{SR}^{w}<J_{DC}^{w}$, Figure 5a).

A similar modeling approach as given by Equation (7) can be used to calculate $J_{SR}^{w}$. If the source for the dissolution process is the solid surface and the sink is the convecting liquid, then the weight fraction of the solid (*w_solid_* = 1), the weight fraction of the dissolution media (*w* ~ 0, sink conditions) and the thickness of the surface reaction layer (*δ_SR_*) are used (Figure 5a). Additionally, the diffusion coefficient must be replaced by a surface reaction coefficient (*k_SR_*) [43], such that

(12) $$J_{SR}^{w}=\frac{k_{SR}\cdot \rho_{solid}}{\delta_{SR}}$$

where the quotient of *k_SR_*∙*δ_SR_*^−1^ represents the mass transfer coefficient due to the surface reaction, that is, the interface velocity. Assuming steady-state, sink conditions and surface-reaction-controlled drug release, the flux depends only on constants, which means it is independent of the relative velocity of the surrounding fluid (Reynolds number).

### 3.4. Model Validation

In order to confirm this model, intrinsic dissolution experiments were performed in the previously described flow channel. Three common model drugs (theophylline, benzocaine and griseofulvin) were chosen based on their different solubilities and UV activities.

**Figure 6:**
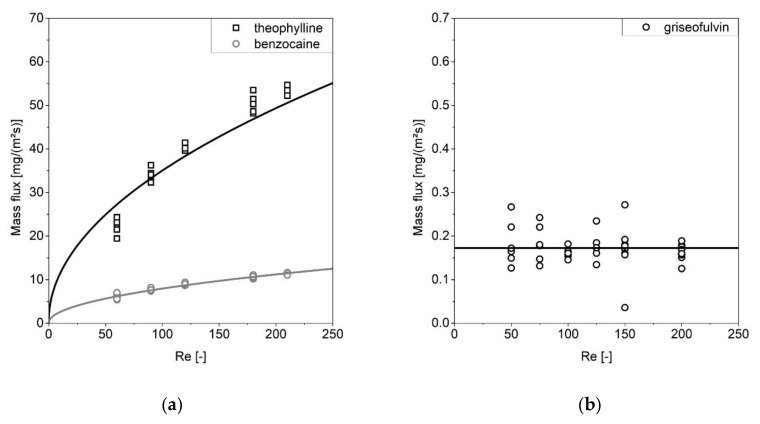
The resulting mass flux of benzocaine, theophylline (**a**) and griseofulvin (**b**) as a function of the Reynolds number.

The results are visualized in Figure 6, where the mass flux is shown with respect to the Reynolds number. Theophylline and benzocaine had an increase in the mass flux with respect to the Reynolds number, which can be attributed to a decrease in the thickness of the diffusion layer (Figure 6a).

Especially for low Reynolds numbers (*Re* < 100), the correlation leads to deviations in the physical data, which is already known from the literature [44]. This might be the reason for the deviation of the *Re* = 60 values for theophylline.

The aforementioned model (Equation (11)) was solved for one single parameter *δ_D_* to describe the experimental data. The diffusion layer thickness at *Re* = 0 (the intercept with the *y*-axis) corresponds to pure diffusion and could be determined for theophylline, 9.79 ± 0.4 mm (av ± CI, α = 0.05), and benzocaine, 5.73 ± 0.1 mm (av ± CI, α = 0.05). Thus, the diffusive mass fluxes of 0.479 mg∙m^−2^∙s^−1^ for theophylline and 0.105 mg∙m^−2^∙s^−1^ for benzocaine could be extracted.

Griseofulvin (Figure 6b) exhibited a mass flux that was independent of the Reynolds number, which is why a surface-reaction-limited mass flux (Equation (12)) was assumed. The particularly high standard deviation within the concentration measurements is remarkable. This is related to the particularly low drug concentration during the measurement, consistent with the low mass flux of 0.172 ± 0.012 mg∙m^−2^∙s^−1^ (av ± CI, α = 0.05).

Overall, it is possible to distinguish between both dissolution mechanisms for drugs and identify suitable formulation concepts. It may be possible to use this technique for formulation and process development as well as to characterize various solid states of the same drug such as polymorphic and amorphous systems.

## 4. Conclusions

The dissolution behavior of drug molecules is limited by either the surface reaction kinetics or the diffusion/convection kinetics. In the absence of convection, the limiting factor is a material property of the drug substance. Therefore, a new dissolution test was developed based on intrinsic dissolution from the pharmacopeia. A flow channel was used to obtain laminar flow conditions with a constant velocity across the whole sample surface.

Computational fluid dynamics was used to determine an appropriate geometry for the flow channel. The apparatus was built and a measuring protocol was developed using different pump speeds to adjust the thickness of the diffusion layer. A model was developed in order to capture experimental data and derive material property data for the surface reaction and diffusion processes.

Three commonly used drug substances were tested, two of which were found to be diffusion limited and one of which was surface reaction limited with respect to the dissolution behavior. The diffusion layer thickness *δ_D_* at *Re* = 0, the corresponding diffusive mass fluxes $J_{D}^{w}$ and the surface reaction mass flux $J_{SR}^{w}$ could be determined reliably, which enabled the extraction of the corresponding mass transfer coefficients. This new technique is a promising tool for identifying beneficial formulation properties based on the dissolution properties of drug substances.

## Data Availability

The data that support the findings of this study are available from the corresponding author upon reasonable request.

## Supplementary Materials

The following are available online at https://www.mdpi.com/1999-4923/13/2/146/s1, Figure S1: Construction drawing of the used flow channel.

## Appendix A

**Table A1:** Symbols used.

Symbol	Description	SI Unit
*A*	area	m^2^
*a*	width	m
*b*	height	m
*c_i_*	concentration of the species i	mol∙m^−3^
*D*	diffusion coefficient	m^2^∙s^−1^
*D_hydr_*	hydrodynamic diameter	m
*J*	molar flux	mol∙m^−2^∙s^−1^
*J^w^*	mass flux per area	kg∙m^−2^∙s^−1^
*J^w^_D_*	diffusive mass flux per area	kg∙m^−2^∙s^−1^
*J^w^_DC_*	diffusive–convective mass flux per area	kg∙m^−2^∙s^−1^
*J^w^_SR_*	surface reaction mass flux per area	kg∙m^−2^∙s^−1^
*k*	Fick’s law coefficient	mol^2^∙s∙kg^−1^∙m^−3^
*k_B_*	Boltzmann constant	kg∙m^2^∙s^−1^∙K^−1^
*k_SR_*	phase transition coefficient	m^2^∙s^−1^
*l_inlet_*	inlet length of the channel	m
*M_i_*	molar mass of the species i	kg∙mol^−1^
*p*	pressure	kg∙m^−1^∙s^−2^
*Re*	Reynolds number	-
*r_mol_*	molar radius	m
*r_sample_*	sample radius	m
*Sc*	Schmidt number	-
*Sh*	Sherwood number	-
*T*	temperature	°K
*U*	circumference	m
$\dot{V}$	volume flow	m^3^
*v*	fluid velocity	m∙s^−1^
*v_max_*	maximum velocity	m∙s^−1^
*w*	weight fraction	-
*w_bulk_*	weight fraction in the bulk liquid	-
*w_s_*	weight fraction at the saturation concentration	-
*w_i_*	weight fraction of the species i	-
*w_solid_*	weight fraction of the solid	-
*x*	coordinate in the axial direction	m
*δ_D_*	diffusion layer thickness	m
*δ_SR_*	interfacial thickness	m
*η*	dynamic viscosity	kg∙m^−1^∙s^−1^
*µ*	chemical potential	-
*ρ_total_*	total density of the binary substance system	kg∙m^−3^
*ρ_solid_*	density of the solute	kg∙m^−3^
*ρ_l_*	density of the liquid	kg∙m^−3^
*λ*	pipe flow resistance	-
