# Supplementary material for: Determination of Inherent Dissolution Performance of Drug Substances
Source: Pharmaceutics. 2021 Jan 22;13(2):146. doi: 10.3390/pharmaceutics13020146 (PMC7911123; doi:10.3390/pharmaceutics13020146)
Supplement: Supplementary file 1 [file pharmaceutics-13-00146-s001.pdf]

pharmaceutics

Supplementary Materials: Determination of Inherent Dissolution Performance of Drug Substances

Dominik Sleziona, Amelie Mattusch, Gerhard Schaldach, David R. Ely, Gabriele Sadowski and Markus Thommes

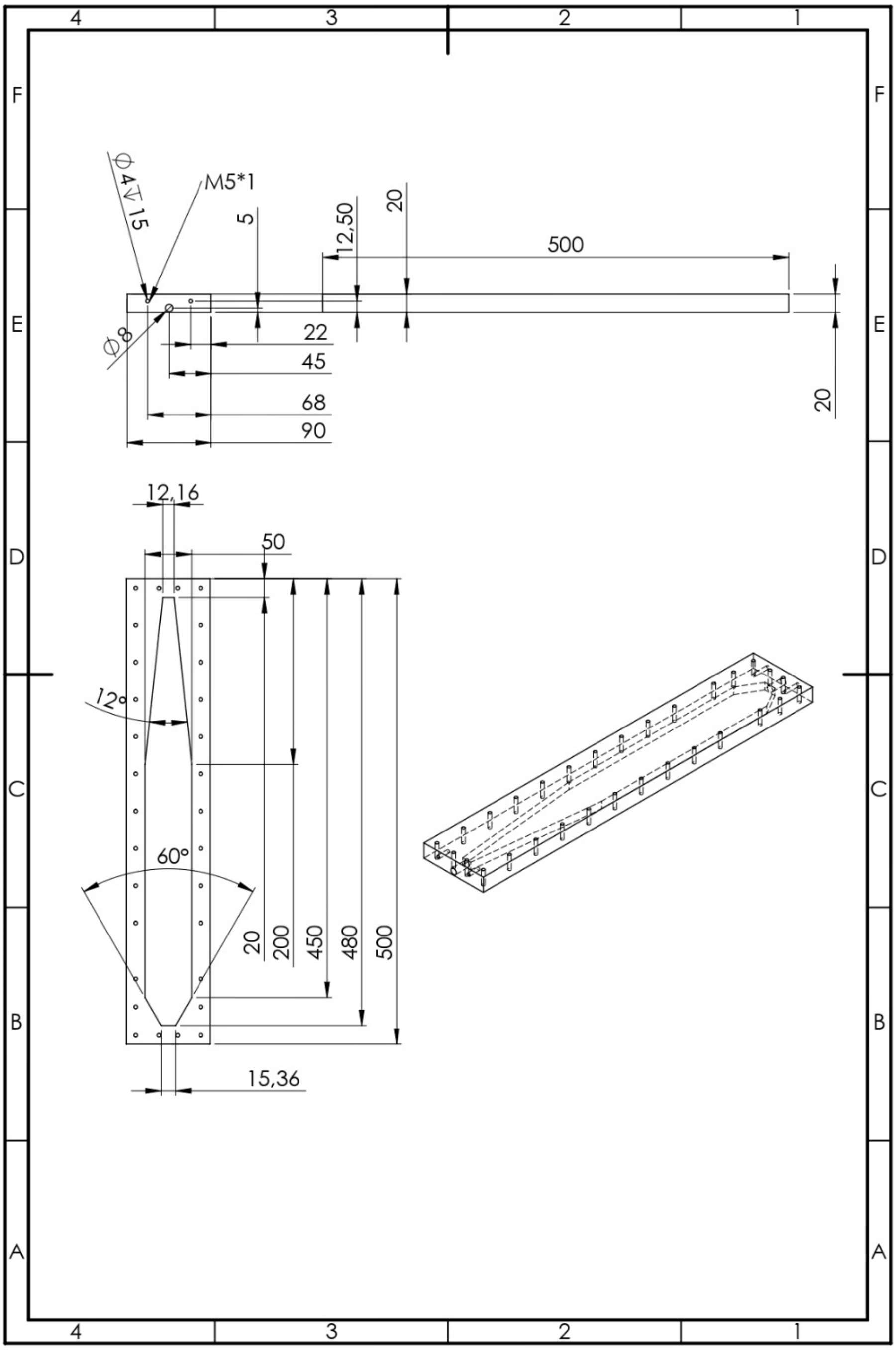

Figure S1 Construction drawing of the used flow channel
